# The Ehlers–Danlos Syndromes against the Backdrop of Inborn Errors of Metabolism

**DOI:** 10.3390/genes13020265

**Published:** 2022-01-29

**Authors:** Tim Van Damme, Marlies Colman, Delfien Syx, Fransiska Malfait

**Affiliations:** Center for Medical Genetics, Department of Biomolecular Medicine, Ghent University, 9000 Ghent, Belgium; tim.vandamme@ugent.be (T.V.D.); marlies.colman@ugent.be (M.C.); delfien.syx@ugent.be (D.S.)

**Keywords:** Ehlers–Danlos syndromes, genetics, pathophysiology, inborn errors of metabolism

## Abstract

The Ehlers–Danlos syndromes are a group of multisystemic heritable connective tissue disorders with clinical presentations that range from multiple congenital malformations, over adolescent-onset debilitating or even life-threatening complications of connective tissue fragility, to mild conditions that remain undiagnosed in adulthood. To date, thirteen different EDS types have been recognized, stemming from genetic defects in 20 different genes. While initial biochemical and molecular analyses mainly discovered defects in genes coding for the fibrillar collagens type I, III and V or their modifying enzymes, recent discoveries have linked EDS to defects in non-collagenous matrix glycoproteins, in proteoglycan biosynthesis and in the complement pathway. This genetic heterogeneity explains the important clinical heterogeneity among and within the different EDS types. Generalized joint hypermobility and skin hyperextensibility with cutaneous fragility, atrophic scarring and easy bruising are defining manifestations of EDS; however, other signs and symptoms of connective tissue fragility, such as complications of vascular and internal organ fragility, orocraniofacial abnormalities, neuromuscular involvement and ophthalmological complications are variably present in the different types of EDS. These features may help to differentiate between the different EDS types but also evoke a wide differential diagnosis, including different inborn errors of metabolism. In this narrative review, we will discuss the clinical presentation of EDS within the context of inborn errors of metabolism, give a brief overview of their underlying genetic defects and pathophysiological mechanisms and provide a guide for the diagnostic approach.

## 1. The Ehlers–Danlos Syndromes, a General Introduction

The Ehlers–Danlos syndromes (EDS) are a group of rare heritable connective tissue disorders, clinically hallmarked by generalized joint hypermobility, skin hyperextensibility and fragility, easy bruising, abnormal wound healing and widespread connective tissue friability [1]. The prevalence is estimated to be approximately 1 in 5000 births. Electron microscopy studies on skin biopsies from affected individuals initially suggested EDS to be a disorder of the collagen ‘wickerwork’ [2], a suspicion that was subsequently confirmed by the biochemical and genetic identification of defects in the primary structure of fibrillar collagens, type I, III and V or their modifying enzymes (lysyl hydroxylase 1 and type I procollagen amino-proteinase) in individuals with different types of EDS [3,4].

These discoveries led to the successful proposal of the Villefranche EDS nosology in 1998, which included six EDS types (classical, hypermobility, vascular, kyphoscoliosis, arthrochalasia and dermatosparaxis) and which was used for over two decades [4]. Meanwhile, advances in molecular techniques identified defects in extracellular matrix (ECM) molecules beyond fibrillar collagens (e.g., the glycoprotein tenascin-X, enzymes involved in proteoglycan biosynthesis, the collagen chaperone FKBP22 and the complement factors C1r and C1s).

This broadened the molecular and clinical spectrum of this syndrome over the past two decades and prompted a revision of the EDS classification [5]. The 2017 International EDS Classification recognizes 13 distinct clinical EDS types, 12 of which have been molecularly elucidated, caused by pathogenic variants in 20 different genes (Table 1) [1,5].

## 2. The Clinical Presentation of EDS, an Overview

Joint hypermobility and skin hyperextensibility are the defining hallmarks of EDS, but a renewed interest in the natural history of these syndromes and the description of several new types has painted an impressive and much more diverse clinical tableau. Depending on the underlying genetic defect, this varies from a mild musculoskeletal and cutaneous phenotype to severe physical disability and life-threatening vascular and visceral complications and can also include such signs and symptoms as skeletal dysplasia, hypotonia, congenital and/or progressive contractures and chronic pain (Table 2).

In general, the autosomal recessive types of EDS are associated with congenital abnormalities, such as hip or other joint dislocations, floppy infant syndrome due to congenital muscle hypotonia and joint hypermobility, kyphoscoliosis, club feet, etc. Although this makes them recognizable entities (very) early on, the diagnosis is often delayed due the fact that EDS is still not a well-known condition.

The diagnosis of the more common and autosomal dominant types (classical (cEDS), hypermobile (hEDS) and vascular EDS (vEDS)) may be missed in childhood due to the presence of only subtle signs of connective tissue fragility that are common in the general population (e.g., joint hypermobility, easy bruising, varicose veins, etc.). This contrasts greatly with most inborn errors of metabolism (IEM) that manifest themselves after a symptom-free period in the first months or years of life [6].

What follows is a concise per system overview of the EDS phenotype keeping a differential diagnosis with the IEM in mind (Table 2).

### 2.1. Prenatal Findings

Prenatal ultrasound abnormalities sometimes seen in e.g., lysosomal storage diseases (hydrops fetalis) or peroxisome biogenesis disorders (brain anomalies and cystic renal disease), are extremely uncommon in EDS [6]. The lone exception are the congenital anomalies of the kidney and urinary tract (nephroptosis and ureteropelvic junction obstruction) sometimes observed in musculocontractural EDS (mcEDS) [7].

There is a higher risk of preterm delivery due to premature rupture of the membranes in some types of EDS and especially in cEDS, vEDS and dermatosparaxis EDS (dEDS), if the fetus is affected [8,9,10].

### 2.2. Integumentary System

**Figure 1:**
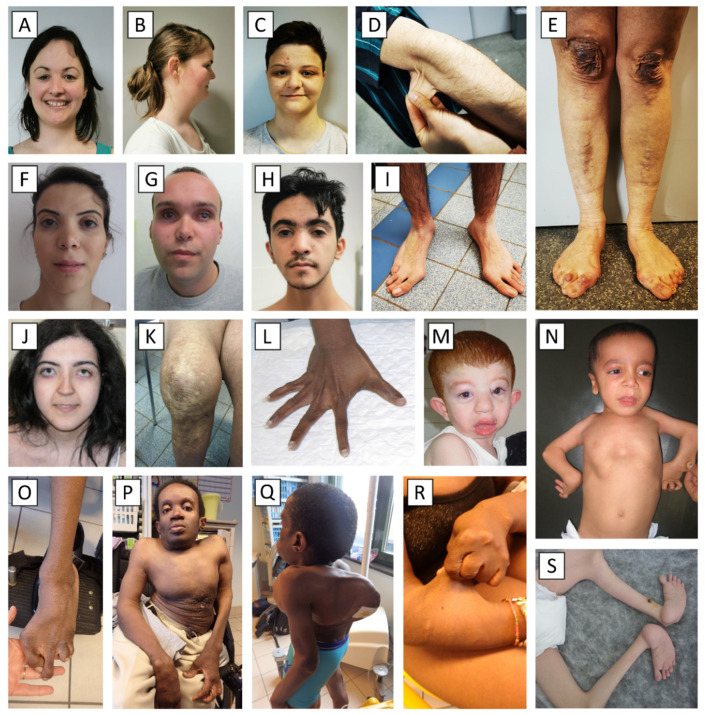
Clinical characteristics of different EDS types. (**A**–**E**): Individuals with classical Ehlers–Danlos syndrome with pathogenic variants in *COL5A1* presenting epicanthal folds (**A**,**C**), a somewhat flattened facial appearance (**B**), skin hyperextensibility (**D**) and atrophic scars and molluscoid pseudotumors (**E**). (**F**–**I**): Individuals with brittle cornea syndrome in whom pathogenic defects in *ZNF469* were identified. Clinical characteristics include hypertelorism, downslanting palpebral fissures, (variable) blue sclerae (**F**–**H**), synophrys (**H**) and deformities of the feet (**I**). (**J**–**L**): Individuals with musculocontractural Ehlers–Danlos syndrome with pathogenic variants in *CHST14* presenting atrophic scars (**K**); facial features including flattened profile, malar hypoplasia, downslanting palpebral fissures, blue sclerae, long philtrum with thin upper lip and protruding jaw with pointed chin (**J**); and characteristic hand deformities (**L**). (**M**): Individual with dermatosparaxis Ehlers–Danlos syndrome and characteristic facial appearance including downslanting palpebral fissures, mild telecanthus, palpebral edema, epicanthic folds, blue sclerae, low-set and floppy ears, saggy cheeks and prominent lips. (**N**–**S**): Individuals with spondylodysplastic Ehlers–Danlos syndrome with pathogenic variants in *B3GALT6* presenting flexion contractures (**N**–**S**), short and deformed extremities (**N**–**S**), muscle atrophy (**S**), severe kyphoscoliosis with pectus deformities (**N**–**Q**) and hyperextensible skin (**R**). Facial features include midfacial hypoplasia with frontal bossing, blue sclerae, downslanting of the palpebral fissures, a short nose with anteverted nares, a long philtrum and low-set ears (**N**,**P**).

The first hallmark of EDS is a hyperextensible skin; i.e., the skin stretches easily and beyond normal but snaps back after release (Figure 1D,R). The skin is often smooth and velvety to the touch. It is fragile and tears easily. This leads to the formation of widened and thin atrophic or so-called ‘cigarette paper scars’, although scar formation may be normal in certain types of EDS (clEDS and pEDS) (Figure 1E,K).

Easy bruising (the spontaneous and recurrent formation of ecchymoses) and hematoma formation are variably present and are most pronounced in vEDS, dEDS, mcEDS and classical-like EDS type 1 (clEDS) [11]. Pretibial hemosiderin deposits (Figure 1D) are a prominent feature of periodontal EDS (pEDS), but can be present in cEDS and vEDS as well. Virtually all dEDS cases reported to date have congenital umbilical hernia. Abdominal herniation (umbilical and inguinal) is occasionally reported in other types of EDS as well [12].

This skin phenotype is markedly different from the one observed in IEM, where pigmentation disorders, lipodystrophy, inverted nipples, aplasia cutis congenita, ichthyosis or dry, thickened scaly skin and xanthomas are observed, all of which are not associated with EDS. The only skin features occasionally shared between rare types of EDS and several types of IEM are hirsutism (dEDS and mcEDS) and a cutis laxa-like skin [13]. The latter is a clinical term for loose, wrinkled, sagging and redundant skin that lacks the excessive elastic recoil typically seen in EDS and can be present particularly in dEDS [10].

### 2.3. Skeletal System

Joint hypermobility, the second hallmark of EDS, is often the reason for referral both in children and adults. Joint hypermobility is a continuous trait modified by age, ethnicity, sex and environmental factors, such as exercise and past traumas. In the general population, generalized joint hypermobility has a reported prevalence of 6% to 57% in females and 2% to 35% in males [14]. (Generalized) joint hypermobility is also observed in other syndromic conditions, including several congenital disorders of glycosylation (e.g., X-linked SSR4-CDG, Morquio syndrome or mucopolysaccharidosis (MPS) type IV) [15].

The severity is routinely assessed using the Beighton scale. Hypermobility is generally defined by a score ≥ 6 for prepubertal children and adolescents, by a score ≥ 5 for pubertal men and women ≤ 50 years of age and by a score ≥ 4 for men and women > 50 years of age. The Beighton scale is easy-to-use, but has some major limitations, and better methods to measure joint mobility in routine clinical practice are needed [5]. Joint dislocations, joint pain and fatigue are complications of generalized joint hypermobility and are reported in all types of EDS [16].

Congenital hip dysplasia is a universal feature in the athrochalasia type of EDS (aEDS), but can be present in other types as well [17]. A marfanoid habitus is not uncommon in some of the rare autosomal recessive types of EDS (kyphoscoliotic EDS (kEDS), mcEDS and brittle cornea syndrome (BCS)) and is contrary to homocystinuria present in combination with joint hypermobility. Osteoporosis is more prevalent in some types of EDS (kEDS, mcEDS, dEDS, BCS and spondylodysplastic EDS (spEDS)) and is associated with an increased risk of fractures, although seldom as pronounced as in osteogenesis imperfecta. This is similar to what is observed in Gaucher disease and homocystinuria.

Other recurring skeletal features include osteoarthritis, spinal and pectus deformities (Figure 1Q), contractures (Figure 1N,O,S), arachnodactyly (Figure 1L) and foot deformations (Figure 1E,I,O,S).

The most severe skeletal phenotype is reported in spEDS [18]. spEDS-*B4GALT7* is predominantly associated with radio-ulnar synostosis, whereas the skeletal findings in spEDS-*SLC39A13* and especially in spEDS-*B3GALT6* are more pronounced and include widening of the metaphyses and bowing of the diaphysis of long bones, hypoplasia of the iliac bones, acetabular dysplasia, femoral head dysplasia and changes to the shape of the vertebral bodies. Spondylodysplastic EDS thus combines the classic signs of EDS with spondyloepimetaphyseal dysplasia, bone fragility and short stature and evokes a wide differential diagnosis that not only includes other types of EDS but also osteogenesis imperfecta, skeletal dysplasias and MPS (e.g., type IV).

### 2.4. Neuromuscular System

Hypotonia is a key symptom of many IEM, but it is less frequent and mostly less severe within the group of the Ehlers–Danlos syndromes. Unsurprisingly, it is most frequently reported in types of EDS with a disorganized myomatrix (clEDS type 1 and myopathic EDS (mEDS)). TNX-deficiency (clEDS type 1) is always associated with some degree of muscle weakness (mild to moderate), myalgia and easy fatigability, but all patients reported to date can walk independently.

The combination of congenital proximal joint contractures with distal joint hypermobility should raise a suspicion of mEDS, which shows clinical overlap with Bethlem myopathy and Ullrich congenital muscular dystrophy and is further characterized by profound congenital hypotonia, which appears to improve with age [19,20].

Hypotonia can also be present in cEDS, aEDS, kEDS, mcEDS and spEDS and is variably associated with delayed gross motor development, myopathy with variability in fiber diameter on muscle biopsy, signs of axonal polyneuropathy on electromyography and elevated creatine kinase [12,21,22].

Central nervous system abnormalities have been reported only in mcEDS-*CHST14* and spEDS-*B3GALT6* and include ventricular enlargement, Dandy–Walker anomalies, hypoplasia of the septum pellucidum, hydrocephalus and a tethered spinal cord [7,12]. Intellectual disability is typically not present but has sporadically been reported in families with AR types of EDS. It remains, however, unclear whether this is truly part of the EDS phenotype or rather caused by environmental or other genetic factors. Encephalopathy is not reported.

### 2.5. Cardiovascular System

Arterial rupture, occurring spontaneously or preceded by aneurysm, arteriovenous fistulae or dissection, is the predominant feature of vEDS. The median survival for vEDS patients is calculated to be 51 years, albeit with a very large range, and most deaths do indeed result from arterial rupture [9,23,24]. Complications are rare in childhood, but 17% will have a first complication by the age of 20 years, and approximately 70% will have had at least one complication by the age of 40 years [23]. Superficial venous insufficiency is also more prevalent and is often early-onset [23].

Vascular complications are, however, not exclusive to vEDS and can be present in other types of EDS as well albeit with important differences in the number, severity and type of complications [11]. The dissection of medium-sized arteries, for instance, has also been reported in several cases of kEDS and to a lesser extent cEDS, and aortic root dilatation has been reported in cEDS and spEDS-*B3GALT6* [8,18]. Another notable example is the risk of intracranial hemorrhage in dEDS and to a lesser extent in mcEDS-*CHST14* and kEDS-*PLOD1*.

This degree of vascular fragility is not seen in IEM; however, recent studies have brought aortic root dilatation to light as an important complication of MPS and more specifically of MPS IVA (Morquio syndrome) [25]. Fabry-like stroke events are not present in EDS.

Severe cardiac valvular disease necessitating surgical valve replacement is the hallmark of the extremely rare cardiac valvular type of EDS (cvEDS), whereas mild valvular disease and especially mitral valve prolapse, might be more prevalent in cEDS, clEDS type 1, kEDS, mcEDS and spEDS [12,26]. Cardiomyopathy has only been reported in a very limited number of cases and is not a consistent finding.

### 2.6. Ocular System

Rupture of the eye globes, either spontaneous or after minor trauma, due to extreme thinning of the cornea is the cardinal feature of brittle cornea syndrome (BCS) and perhaps one of the most debilitating complications within the EDS spectrum [12,27]. It frequently occurs at a young age; however, several adults without ocular rupture have been described. Prior to rupture, corneal thinning will manifest itself by the presence of blue sclerae and corneal ectasia.

In the other types of EDS there is no or a relatively mild ophthalmic phenotype that includes keratoconus (vEDS), blue sclerae (aEDS, dEDS, kEDS, mcEDS and spEDS), microcornea (kEDS-*PLOD1* and mcEDS) and rarely corneal clouding (spEDS). The latter is more frequently encountered in IEM, such as MPS, Fabry disease, tyrosinemia type II, cystinosis, mucolipidosis IV and Niemann–Pick disease type A. Refractive errors also appear to be more prevalent in EDS compared to the general population.

### 2.7. Orocraniofacial

A recognizable facial gestalt has been reported in only a few types of EDS. Perhaps best known is the thin vermilion of the lips, micrognathia, narrow nose, prominent eyes and potentially severe gingival recession, which can lead to tooth loss in some individuals with vEDS [9]. The latter should also raise the concern of the periodontal form of EDS, in which early-onset periodontitis with extensive periodontal destruction and loss of teeth, starting in childhood or adolescence, is universally present and can be accompanied by similar craniofacial dysmorphisms [28].

The facial dysmorphisms in dEDS also combine into a recognizable appearance and include prominent and protuberant eyes with puffy, edematous eyelids and excessive periorbital skin, large fontanels and/or wide cranial sutures, micrognathia and gingival hyperplasia (Figure 1M) [10]. Finally, individuals with mcEDS present with large fontanels, downslanting palpebral fissures, hypertelorism, a short nose with hypoplastic columella, ear deformities, high-arched palate, long philtrum, thin upper lip vermilion, small mouth and microretrognathia (Figure 1J) [7,29].

Several other types of EDS are associated with less specific findings, such as epicanthal folds (Figure 1A–C) (cEDS, aEDS and kEDS), frontal bossing (Figure 1F–H) (aEDS, spEDS-*B3GALT6* and BCS) and hypertelorism (Figure 1F–H,J) (aEDS, mcEDS, kEDS and BCS), etc. [12].

### 2.8. Other

The above is only a curated selection from a much more extensive list of anomalies, symptoms and signs described in EDS. To conclude, we will highlight a few clinical pearls that might facilitate early diagnosis.

In addition to vascular rupture, gastrointestinal perforation, usually involving the sigmoid, or hollow organ rupture can be the presenting signs in vEDS [23,30]. Spontaneous pneumothorax is most often associated with Marfan syndrome and Birt–Hogg–Dubé syndrome but is also seen in ~10% of individuals with vEDS [9]. 

Hearing loss is a key finding in heritable connective tissue disorders, such as osteogenesis imperfecta and Stickler syndrome but is also present in the majority of individuals with kEDS-*FKBP14* or mcEDS and a third of the individuals with BCS [7,12,27]. Finally, cryptorchidism is present in the large majority of mcEDS-*CHST14* individuals and should raise suspicion of mcEDS in a neonate with signs of connective tissue fragility and contractures [7].

## 3. The Genetic and Pathophysiological Basis of the EDS

**Figure 2:**
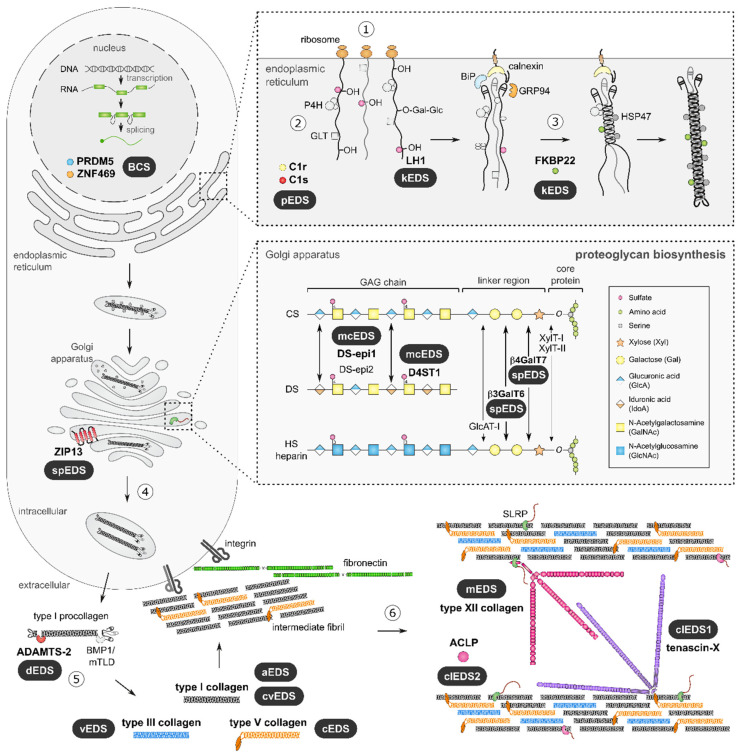
Collagen and proteoglycan biosynthesis in the context of EDS. Defective molecules associated with EDS are indicated in bold, and the respective EDS type is indicated. Fibrillar collagen biosynthesis is initiated by transcription and translation of pro-α-chains (step 1). Nascent pro-α-chains are co- and post-translationally modified by several modifying enzymes in the endoplasmic reticulum (ER), such as proline and lysine hydroxylases and galactosyltransferases (step 2). Triple helix formation starts by the association of the C-terminal propeptides of three pro-α-chains and propagates towards the N-terminus in a zipperlike fashion during which several molecular chaperones assist (step 3). Trimeric procollagen molecules aggregate laterally, are transported in secretory vesicles and are secreted into the extracellular environment (step 4). Collagen molecules are formed by removal of the N- and C-propeptides by ADAMTS-2 and BMP-1/mTLD, respectively (step 5). These collagens subsequently assemble into highly ordered striated fibrils. The assembly of collagen fibrils is tissue-specific and requires several assisting proteins (step 6). Fibronectin and integrins serve as *organizers* of fibril assembly at the plasma membrane. At the cell surface, some collagens, including type V collagen, function as *nucleators* and initiate immature fibril assembly. Type V collagen co-assembles with type I collagen to form heterotypic fibrils with the entire triple helical domain of type V collagen embedded within the fibril. The partially processed N-propeptide domain of type V collagen protrudes to the fibril surface where it controls fibrillogenesis by sterically hindering the addition of collagen monomers. Intermediate fibrils are deposited into the ECM and stabilized by interactions with *regulators*, such as the small leucine-rich proteoglycan (SLRP) decorin, tenascin-X and type XIII collagen. These molecules influence the rate of assembly, size and structure of the collagen fibrils. Subsequent fibril growth occurs through linear and lateral fusion of intermediate collagen fibrils, which are stabilized by intra- and inter-molecular crosslinks. Proteoglycan biosynthesis is initiated by the synthesis of a core protein, which is then modified by several Golgi-resident enzymes. First, a common linker region in formed by the addition of four monosaccharides. Formation of this tetrasaccharide linker region begins with the stepwise addition of a xylose (Xyl) residue to a serine residue of the core protein, catalyzed by xylosyltransferase-I and -II (XylT-I/-II). Subsequently, two galactose (Gal) residues are added by galactosyltransferase-I (GalT-I or β4GalT7) and galactosyltransferase-II (GalT-II or β3GalT6). Finally, the addition of a glucuronic acid (GlcA), catalyzed by glucuronosyltransferase-I (GlcAT-I) completes the formation of the linker region. The alternating addition of either N-acetylglucosamine (GlcNAc) or N-acetylgalactosamine (GalNAc) and GlcA to the nascent GAG-chain result in the formation of heparan sulfate (HS) proteoglycans and chondroitin sulfate (CS)/dermatan sulfate (DS) proteoglycans. The GAG-chains are further modified by epimerization and sulfation reactions. The epimerization of GlcA towards iduronic acid (IdoA), which is catalyzed by DS epimerases-1 and -2 (DS-epi1 and DS-epi2) is necessary for the formation of DS. Subsequently, dermatan 4-*O*-sulfotransferase 1 (D4ST1) is able to catalyze 4-*O*-sulfation of GalNAc, which prevents back-epimerization of the adjacent IdoA.

The pleiotropic and multisystemic nature of EDS is caused by alterations in the physical properties of the ECM in many tissues and organs, which, in turn, is the result of a compromised biosynthesis, fibrillogenesis and/or supramolecular organization of collagen fibrils due to pathogenic variants in different genes (Figure 2).

The biosynthetic pathway of fibrillar procollagens is a complex process. Nascent pro-α-chains are heavily post-translationally modified. Three pro-α-chains associate at their carboxy-(C-) terminal propeptides and assemble into a trimeric procollagen molecule propagating to the amino-(N-) terminus in a zipper like fashion. These trimeric proteins consist of either three identical (homotrimer) or genetically distinct (heterotrimer) pro-α-chains, containing repeating Gly--Xaa--Yaa triplets of a glycine residue and two other amino acids and which form a long uninterrupted triple helical domain flanked by globular C- and N-terminal propeptides.

These procollagen molecules are subsequently transported to the extracellular environment. Proteolytic removal of the N- and C-propeptides results in the formation of a mature collagen molecule that can assemble into highly ordered cross-striated fibrils and fibers [31].

Typically, collagen fibrils are composed of different fibrillar collagen types, e.g., collagen I and collagen III (heterotypic collagen I/III fibrils) and the assembly of collagen fibrils occurs in a tissue-specific way, requiring the concerted action of several assisting proteins: organizers (e.g., fibronectin and integrins), nucleators (e.g., type V collagen) and regulators (e.g., small leucine-rich proteoglycans (SLRPs), fibril-associated collagens with interrupted triple helices (FACIT) and glycoproteins) [32].

During fibrillogenesis, fibril growth occurs through linear and lateral fusion of intermediate collagen fibrils, which are subsequently stabilized by the formation of intra- and inter-molecular crosslinks (Figure 2) [33,34,35]. The EDS types can be grouped based on the pathways in which the defective proteins function (Table 1) [5].

### 3.1. Defects in Fibrillar Collagen Structure and Processing

The first biochemical and/or molecular defects associated with EDS were shown to result from defects in the primary structure, processing, or modification of the fibrillar procollagen types I, III and V leading to qualitative and/or quantitative changes in these collagen types.

**Classical EDS**—The classical type of EDS (cEDS) is caused by heterozygous pathogenic variants in the *COL5A1* or *COL5A2* genes, encoding the pro-α1- and pro-α2-chains of type V collagen, respectively. Type V collagen is a low abundant protein in skin, tendons and bone. It is mostly present as a heterotrimer consisting of two pro-α1(V)-chains and one pro-α2(V)-chain, which assembles with type I collagen to form heterotypic type I/V collagen fibrils, where it plays a crucial role during the initiation and regulation of collagen fibrillogenesis [33,36]. Central to the pathogenesis of cEDS is a decreased amount of functional type V collagen in the ECM.

**Figure 3:**
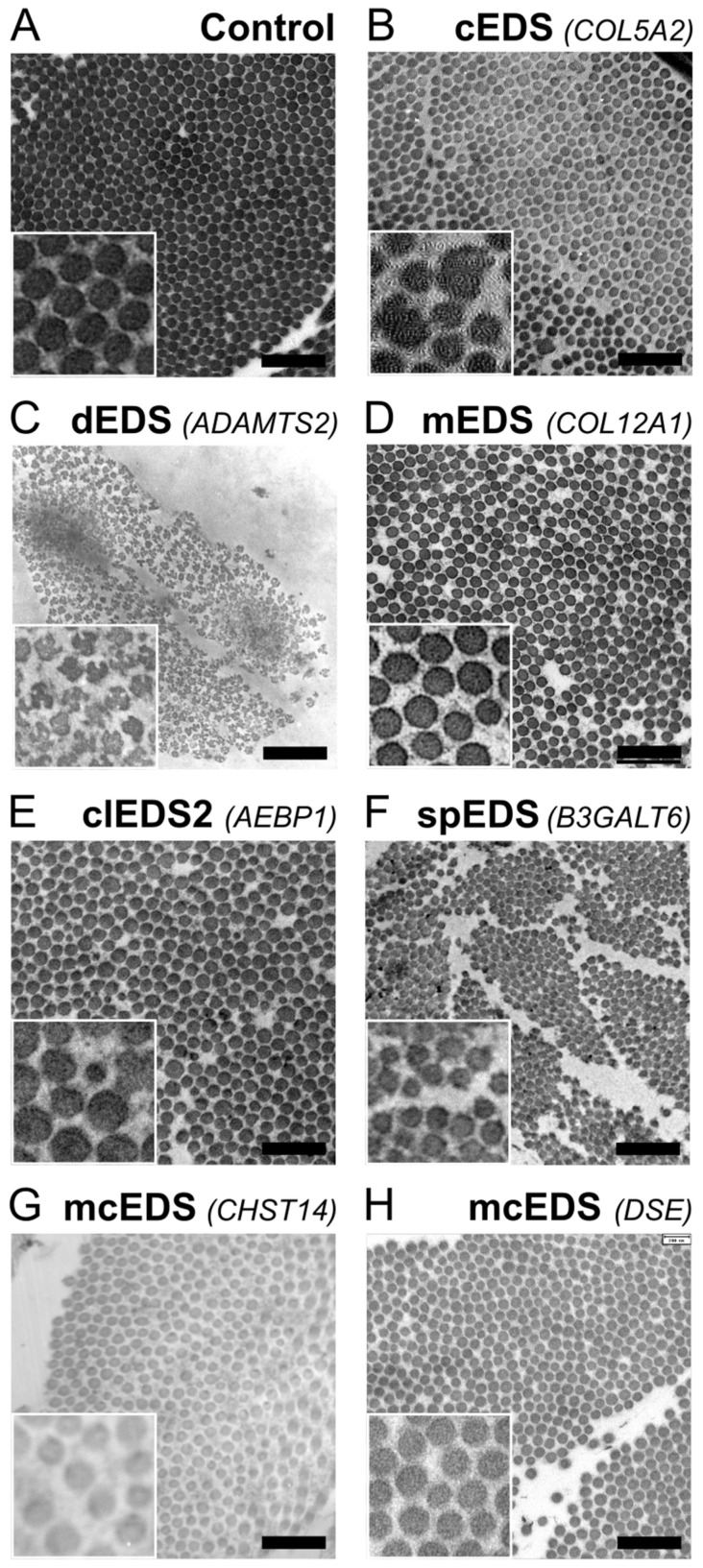
Ultrastructural findings of dermal collagen fibrils in different EDS types. (**A**) Control showing tightly packed collagen fibrils with uniform diameters. (**B**) Presence of some very large fibril diameters with irregular contours, called ‘collagen cauliflowers’ in classical EDS (cEDS) due to a heterozygous pathogenic variant in *COL5A2*. (**C**) Hieroglyphic aspect of the collagen fibrils in dermatosparaxis EDS (dEDS) due to biallelic pathogenic variants in *ADAMTS2*. (**D**) Increased interfibrillar spacing myopathic EDS (mEDS) due to a heterozygous pathogenic variant in *COL12A1*. (**E**) Collagen fibrils with large and small collagen fibril diameters and irregular contours in classical EDS-like type 2 (clEDS2) due to biallelic pathogenic variants in *AEBP1*. (**F**) Dispersed collagen fibrils with variable collagen fibril diameters, sporadic fibrils with very irregular contours and granulofilamentous deposits between collagen fibrils in spondylodysplastic EDS (spEDS) due to biallelic pathogenic variants in *B3GALT6*. (**G**) Collagen fibrils with variable diameters and the intermittent presence of small flower-like fibrils and irregular interfibrillar spaces filled with granulofilamentous deposits in musculocontractural EDS (mcEDS) due to biallelic pathogenic variants in *CHST14*. (**H**) Grossly normal collagen fibril architecture with mildly increased interfibrillar spacing in musculocontractural EDS (mcEDS) due to biallelic pathogenic variants in *DSE*. Scale bars: 500 nm.

Ultrastructural analysis using transmission electron microcopy (TEM) of skin biopsies from cEDS patients revealed the presence of atypical (cauli)flower-shaped collagen fibrils on cross-section (Figure 3B).

Nevertheless, the molecular consequences remain poorly understood. Studies on dermal fibroblasts from cEDS patients did show decreased migration capacity and alterations in the deposition and organization of several ECM molecules, as well as changes in specific integrin receptors [37]. Subsequent transcriptome-wide analysis on cEDS dermal fibroblasts confirmed a deregulation of genes involved in ECM remodeling and wound healing and indicated a role for altered endoplasmic reticulum (ER) homeostasis and autophagy [38].

**Vascular EDS**—Vascular EDS (vEDS) is caused by heterozygous pathogenic variants in the *COL3A1* gene encoding the pro-α1-chain of type III collagen. The homotrimeric type III collagen is mainly found in tissues with elastic properties (e.g., skin, blood vessels, gastrointestinal tract, uterus etc.) and can also assemble with type I collagen to form heterotypic fibrils.

Like type V collagen, type III collagen is presumed to regulate collagen fibril assembly and diameter [39,40]. Transcriptome profiling of dermal fibroblasts from vEDS patients revealed differential expression of genes encoding ECM molecules as well as genes involved in ER homeostasis [41]. Ultrastructural studies on the skin from vEDS patients showed a thinner dermis, aberrant diameters and distribution of collagen fibrils and variable dilatation of the ER [42].

**Type I collagen defects**—While most pathogenic defects in the pro-α1- and pro-α2-chains of type I collagen, encoded by the *COL1A1* and *COL1A2* genes, respectively, result in the brittle bone disorder osteogenesis imperfecta, some specific defects are associated with rare EDS types.

Heterozygous pathogenic variants resulting in partial or complete deletion of exon 6 of the *COL1A1* or *COL1A2* gene result in the arthrochalasia type of EDS (aEDS). Exon 6 of these genes contains the cleavage site for the N-propeptide and its loss leads to partial processing of type I procollagen with retention of the N-propeptide of either the pro-α1(I)- or the pro-α2(I)-chain in the collagen fibrils [43].

Decreased N-propeptide cleavage of the type I procollagen N-propeptide can also be caused by defects in the type I procollagen N-proteinase, ADAMTS2, due to biallelic loss-of-function variants in the *ADAMTS2* gene, which gives rise to the dermatosparaxis EDS type (dEDS). In contrast to aEDS, where either the pro-α1(I)-or the pro-α2(I)chain is affected, cleavage of both pro-α(I)-chains is compromised in dEDS. ADAMTS2 is also known to possess N-proteinase activity for types II, III and V pro-collagens and enzymatic activity for some other molecules [44,45], which likely contributes to the phenotypic differences between aEDS and dEDS.

Incorporation of incompletely processed collagen molecules (still containing N-propeptides) leads to ultrastructural abnormalities of the dermal collagen fibrils, ranging from small-diameter fibrils with irregular contours in aEDS to fibrils that have completely lost their circular dimensions on cross-sections and display a typical hieroglyphic pattern in dEDS (Figure 3C) [10,46].

Biallelic loss-of-function variants in the *COL1A2* gene, that lead to unstable mRNA prone to nonsense-mediated mRNA decay and complete lack of the pro-α2(I)-chains, cause the extremely rare cardiac-valvular EDS (cvEDS). The absence of pro-α2(I)-chains forces the formation of α1(I) homotrimers; however, the exact pathomechanisms remain elusive.

Finally, specific heterozygous variants in the *COL1A1* gene that substitute an arginine for a cysteine residue in the triple helical region of the pro-α1(I)-chains give rise to a rare phenotype clinically overlapping with cEDS and vEDS [47,48]. In addition to the local destabilization of the type I collagen molecules due to the loss of the helical arginine residue, the introduction of a cysteine residue results in the production of α1(I) dimers and dermal collagen fibrils have variable diameters with irregular interfibrillar spaces [47].

### 3.2. Defects in Collagen Crosslinking and Folding

**Kyphoscoliotic EDS-*PLOD1***—The first inborn error of collagen metabolism that was elucidated at the biochemical level was kyphoscoliotic EDS (kEDS) with the demonstration of a reduced hydroxylysine content in the dermal collagen of two affected sisters, caused by a lysyl hydroxylase deficiency [49,50]. Subsequently, biallelic pathogenic variants in the *PLOD1* gene encoding the collagen modifying enzyme lysyl hydroxylase 1 (LH1) were identified [51]. 

LH1 catalyzes the co- and posttranslational modification of specific lysine residues in the triple helix to hydroxylysine. These hydroxylysyl residues can either be glycosylated or can form intermolecular crosslinks in the ECM. As a consequence of LH1 deficiency, abnormal crosslink formation in kEDS-*PLOD1* patients renders their tissues mechanically instable.

**Kyphoscoliotic EDS-*FKBP14***—More recently, biallelic variants in the *FKBP14* gene were found to cause a form of EDS that shows phenotypic overlap with kEDS-*PLOD1*. *FKBP14* encodes the ER-resident protein FKBP22 (FK506 Binding Protein 22 kDa), a peptidyl-prolyl *cis-trans* isomerase that accelerates procollagen folding in vitro [52,53]. In addition, FKBP22 functions as a molecular chaperone and preferentially interacts with type III, IV, VI and X procollagen but does not bind to type I or V collagen [52,54]. Dermal fibroblasts from FKBP22-deficient patients show enlarged ER, likely reflecting the accumulation of collagen in the ER, as well as altered in vitro assembly of the ECM [55].

Transcriptome profiling of dermal fibroblast cultures of kEDS-*PLOD1* and kEDS-*FKBP22* patients showed distinct transcriptome signatures with differentially expressed ECM components, which are unique to each kEDS type [56]. The pathophysiological mechanisms linking kEDS-*PLOD1* and kEDS-*FKBP14* remain currently unknown.

### 3.3. Defects in ECM Bridging Molecules

**Classical-like EDS type 1**—The first glycoprotein involved in EDS pathogenesis was tenascin-X (TNX). Biallelic loss-of-function variants in the *TNXB* gene were shown to lead to a phenotype similar to that caused by defects in type V collagen; hence, this EDS type was coined classical-like EDS (clEDS) [57,58].

The *TNXB* gene partially overlaps with the *CYP21A2* gene, encoding 21-hydroxylase. Deficiency of 21-hydroxylase leads to congenital adrenal hyperplasia (CAH). A contiguous gene syndrome that combines signs of CAH and signs of EDS that is caused by deletions in CYP21A1 that extend into TNXB has been described [59]. Additionally, in a subset of individuals presenting generalized joint hypermobility, heterozygous *TNXB* variants were identified that lead to *TNXB* haploinsufficiency [60].

**Myopathic EDS**—Biallelic and heterozygous defects in the *COL12A1* gene, encoding the pro-α1-chains of type XII collagen, cause the rare myopathic EDS (mEDS).

The homotrimeric type XII collagen is a member of the FACIT family and interacts with TNX [61]. Both molecules can directly or indirectly (e.g., via SLRPs, such as decorin or fibromodulin) bind to fibrillar collagens and form flexible bridges between collagen fibrils and other ECM molecules, thereby, regulating the organization and mechanical properties of collagen fibrils. This is supported by ultrastructural studies of dermal collagen fibrils of both clEDS1 and mEDS patients showing increased interfibrillar space (Figure 3D) [62,63].

**Classical-like EDS type 2**—More recently, biallelic pathogenic variants were identified in the *AEBP1* gene in a few individuals also presenting a phenotype that strongly overlaps with cEDS and clEDS type 1. *AEBP1* codes for adipocyte enhancer-binding protein 1 (AEBP1; also known as aortic carboxypeptidase-like protein, ACLP) [64]. AEBP1 is an ECM-associated protein that occurs in tissues with a high collagen content [65,66], binds to type I, III and V collagen, and aids type I collagen polymerization [64].

Ultrastructural studies of patients with biallelic *AEBP1* variants revealed dermal collagen fibrils with variable diameters and irregular contours, suggesting the involvement of AEBP1 in collagen fibril formation (Figure 3E) [67]. Furthermore, roles for AEBP1 have been reported in fibroblast-to-myoblast transition via TGFβ-signaling [68] and frizzled-8- and LRP6-mediated activation of canonical Wnt-signaling. [69]. This condition is provisionally referred to as classical-like EDS type 2 (clEDS2).

### 3.4. Glycosaminoglycan Biosynthesis

Proteoglycans are major ECM constituents that play crucial structural roles and are involved in modulation of cell adhesion and motility, assembly of collagen fibrils and ECM as well as signal transduction. Proteoglycans consist of a core protein containing one or more glycosaminoglycan (GAG)-chains [70,71,72]. The biosynthesis of these GAG chain starts with the addition of four sugar residues to the core protein to form a tetrasaccharide linker, followed by the addition of repeating disaccharide units that define the GAG-chain as heparan sulfate (HS), chondroitin sulfate (CS) and/or dermatan sulfate (DS) (Figure 2).

**Spondylodysplastic EDS**—The first involvement of proteoglycan defects in EDS pathogenesis was reported in the 1980s with the discovery of GAG-free proteoglycan core proteins produced by fibroblasts of a patient with a condition coined ‘progeroid EDS’ [73,74,75,76,77]. Later, biallelic variants were identified in the *B4GALT7* gene, encoding galactosyltransferase-I, the enzyme catalyzing the addition of the second sugar residue during the formation of the tetrasaccharide linker region of proteoglycans [78].

Subsequently, in another form of EDS, biallelic defects were discovered in the *B3GALT6* gene, encoding galactosyltransferase-II, catalyzing the addition of the second galactose residue (i.e., the third step) during linker formation. Both conditions are now labelled spondylodysplastic EDS (spEDS). Defective galactosyltransferase-I or -II activity results in variable reduction in HS, CS and DS contents on proteoglycans. Interestingly, studies on *b3galt6* knockout zebrafish demonstrated the presence of an unconventional trisaccharide linker region consisting of only three sugar residues instead of four [79].

**Musculocontractural EDS**—In addition to alterations in linker formation, defects in enzymes specifically responsible for modifying DS GAG-chains have also been linked to EDS pathogenesis. Musculocontractural EDS (mcEDS) is caused by biallelic loss-of-function variants in either the *CHST14* or *DSE* gene, encoding dermatan 4-*O*-sulfotransferase-1 (D4ST1) and dermatan sulfate epimerase 1 (DS-epi1), respectively [80,81,82,83]. These enzymes catalyze consecutive steps in DS biosynthesis (Figure 2), and their deficiency leads to either complete replacement of DS for CS in D4ST1-deficient patients or severely reduced DS moieties in DS-epi1-deficient patients [83,84].

Altered or lacking GAG chains result in proteoglycans, such as the SLRP decorin, that are not capable of executing their crucial function during collagen fibrillogenesis, hence, leading to abnormal organization of collagen fibrils in the dermis (Figure 3F–H) of individuals with mcEDS or spEDS due to *B4GALT7* or *B3GALT6* defects.

### 3.5. Defects in Intracellular Processes

**Spondylodysplastic EDS-*SLC39A13***—A very small subset of EDS patients harbor biallelic pathogenic variants in the *SLC39A13* gene, encoding the homodimeric transmembrane Zrt/irt-like protein 13 (ZIP13), which regulates zinc influx in the cytosol. ZIP13 deficiency results in uniform underhydroxylation of lysine and proline residues in collagen and abnormal collagen crosslinking in the ECM [85]. 

The proposed disease mechanisms include competition of increased Zn^2+^ levels in the ER with Fe^2+^, a cofactor for lysyl and prolyl hydroxylases [85], vesicular Zn^2+^ trapping in the ER resulting in decreased Zn^2+^ availability [86] and altered activation of BMP/TGFβ signaling [87]. Due to the clinical overlap, this condition is merged with spEDS (spEDS-*SLC39A13*); however, the mechanisms linking spEDS-*B4GALT7*, spEDS-*B3GALT6* and spEDS-*SLC39A13* remain elusive.

**Brittle cornea syndrome**—Initially suspected to be a form of kEDS, brittle cornea syndrome (BCS) was demonstrated to be caused by biallelic pathogenic variants in either the *ZNF469* or the *PRDM5* gene [88,89,90]. *ZNF469* encodes the zinc finger protein ZNF469 for which the function is largely unknown. *PRDM5* encodes the transcription factor PRDM5 (PR domain zinc finger protein 5), which modulates several facets of vertebral tissue development and maintenance and is dependent on Wnt signaling [91,92]. 

Transcriptome analysis of dermal fibroblasts obtained from patients with pathogenic variants in *ZNF469* or *PRDM5* demonstrate altered expression of genes involved in ECM regulation. Additionally, immunofluorescent analysis showed altered in vitro deposition of ECM molecules (e.g., type I, III collagen and fibronectin) and some integrin receptors on dermal fibroblasts of BCS patients [89,93]. These overlapping findings point to a role for ZNF469 and PRDM5 in regulating the organization of the ECM.

### 3.6. Defects in the Complement Pathway

**Periodontal EDS**—Recently, heterozygous pathogenic gain-of-function variants in the *C1R* and *C1S* genes, encoding the C1r and C1s subunits of the first component of the classical complement pathways, were associated with periodontal EDS (pEDS) [28]. Intracellular activation of C1r and/or C1s and the extracellular presence of activated C1s, which can, in turn, activate the classical complement cascade, are central to pEDS pathogenesis [94]. 

Furthermore, the presence of evolutionarily conserved CUB domains in C1r and C1s suggest that they can also interact with ECM molecules including collagens and/or propeptides [95]. These findings provide opportunities to gain more knowledge about the connection between connective tissues and the immune system.

## 4. Diagnostic Approach

The 2017 International EDS Classification defined major and minor clinical criteria to guide the diagnosis of the different types of EDS [5]. Nevertheless, the definite diagnosis for all EDS types, except for hEDS, relies on the identification of a molecular defect in the respective genes. For hEDS, the genetic basis remains unknown, and diagnosis relies solely on proper clinical and familial evaluation [5].

The currently used diagnostic workflow mainly applies next-generation sequencing-based approaches focusing on parallel sequencing and subsequent analysis of a panel containing (at least) the 20 known EDS genes, to obtain a time- and cost-effective genetic diagnosis. This analysis should be complemented by a strategy to detect genomic copy number variants, such as array comparative genomic hybridization or shallow whole-genome sequencing.

Special care should be taken when analyzing certain genes. The *TNXB* gene has a partially duplicated non-functional pseudogene, *TNXA*, which shares 97% identity to the 3′ region of *TNXB*. Therefore, exons 32–44 of *TNXB* should be analyzed using a Sanger sequencing-based method [5]. An 8.9 kb duplication of seven exons (exon 10–16) is the most common pathogenic variant in *PLOD1*, being found in ~30% of kEDS-*PLOD1* cases, and can be detected using a specifically designed PCR or via Multiplex ligation-dependent probe amplification (MLPA).

Despite rigorous genetic testing, there are still patients that present with a clinical EDS diagnosis (hEDS but also other EDS-like phenotypes) in whom no causal genetic defect can be found. This can either be attributed to technical limitations (e.g., deep intronic variants, variants in promotor or untranslated regions (UTRs)) or can point to additional genetic heterogeneity, with novel, yet to be discovered disease genes.

For several EDS types, biochemical and/or ultrastructural analyses can be performed, but they require a skin biopsy, urine or a blood sample. Although several of these analyses were once part of the routine diagnostic work-up, they are now mainly relegated to study the pathogenicity if variants of uncertain significance (VUS) are found, if only one pathogenic variant is detected in a recessive type of EDS or if no pathogenic variants could be detected in a patients with a clear clinical EDS diagnosis.

### 4.1. Biochemical Analyses

**(Pro)collagen analysis**—Sodium dodecyl sulfate polyacrylamide gel electrophoresis (SDS-PAGE) of radioactively or fluorescently labeled (pro)collagens isolated from cell and conditioned medium fractions of dermal fibroblast cultures established from a skin biopsy of EDS patients can show quantitative (reduced amounts) and/or qualitative (intracellular retention or altered electrophoretic mobility) alterations in type I and III procollagen. The (pro-)α-chains are partially digested with pepsin (cell and medium fraction) to remove the N- and C-propeptides or left untreated (procollagen analysis) prior to SDS-PAGE. 

**Figure 4:**
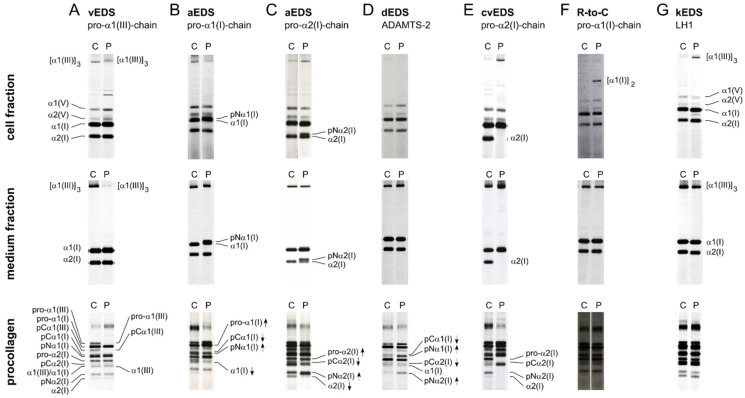
Illustration of representative (pro)collagen electrophoretic mobility patterns for different EDS types. Metabolically labelled (pro)collagen chains isolated from cells and conditioned medium of dermal fibroblast cultures were either partially digested with pepsin (cell and medium fraction) or left untreated (procollagen) prior to SDS-PAGE. All individual intermediate and mature (pro)collagen chains are indicated on the left side of panel A as a reference. At the right side of the gels, (pro)collagen chains displaying a difference are highlighted. pNα- and pCα-chain denote pro-α-chains that contain only the N- or C-terminal propeptide, respectively. C: control and P: patient. (**A**) Severely reduced amounts of the type III (pro)collagen homotrimer in the cell and medium fractions in a vascular EDS (vEDS) patient. (**B**) The presence of additional mutant pNα1(I)-chains, in the cell and medium fractions of patients with arthrochalasia EDS (aEDS) caused by a defect in *COL1A1*. (**C**) The presence of additional mutant pNα2(I)-chains, in the cell and medium fractions of patients with arthrochalasia EDS (aEDS) caused by a defect in *COL1A2*. (**D**) Accumulation of procollagen chains with a retained N-propeptide (pNα1(I) and pNα2(I)) and nearly complete absence of bands representing the pCα1(I) and pCα2(I) procollagen chains in a patient with dermatosparaxis EDS (dEDS) due to biallelic *ADAMTS2* defects. Of note, a normal electrophoretic mobility of the collagen chains is seen in the cell and medium fractions because propeptides are enzymatically removed with pepsin during sample preparation. (**E**) Complete absence of the α2(I) procollagen chains in cell and medium fractions in a patient with cardiac-valvular EDS (cvEDS). (**F**) Abnormal disulfide-bonded α1(I) dimers are present in the cell layer but not in the medium fraction of a patient with a pathogenic variant in the *COL1A1* gene leading to an arginine-to-cysteine substitution in the α1(I)-chain (c.934C>T, p.(Arg312Cys)). (**G**) Type I, III and V (pro)collagen chains from a kyphoscoliotic EDS (kEDS) patient with biallelic *PLOD1* mutations show a uniformly faster migration in both cell and medium fractions and on procollagen gels, thereby, demonstrating underhydroxylation and underglycosylation of lysyl residues.

Qualitative or quantitative alterations in type III (pro)collagen can be detected in about 95% of vEDS patients (Figure 4A). EDS-related defects affecting type I collagen can also be detected. SDS-PAGE analysis of pepsin-digested collagens from aEDS fibroblasts shows an extra band containing the retained N-propeptide, the co-called pNα-chain, in addition to the normal α1(I)- or α2(I)-chains (Figure 4B,C). In contrast, fibroblasts from dEDS patients show an accumulation of type I procollagen chains with a retained N-propeptide when analyzing the procollagen fraction (Figure 4D). 

Complete absence of the (pro-)α2(I)-chains is indicative of cvEDS (Figure 4E), and the presence of an additional band in cell and/or medium fractions, representing α1(I) dimers, can point to the presence of specific arginine-to-cysteine substitutions in the pro-α1(I)-chain (Figure 4F). Furthermore, uniform underhydroxylation and underglycosylation of the (pro-)α-chains as a consequence of LH1 deficiency in EDS-*PLOD1* patients, can be seen as a faster migration of type I, III and V (pro)collagen chains (Figure 4G). (Pro)collagen analysis of type V collagen is not very sensitive to pick up defects in type V collagen [96].

**Urinary analyses**—Quantification of lysylpyridinolines (LP) and hydroxylysylpyridinolines (HP) crosslinks in urine using high-performance liquid chromatography is a reliable test to identify LH1 defects. The normal LP/HP ratio in healthy individuals is 0.2, whereas kEDS-*PLOD1* patients have increased ratios ranging from 2 to 9 [97]. A mildly increased LP/HP ratio of around 1 is seen in patients with spEDS caused by pathogenic variants in *SLC39A13* [85].

Defective DS biosynthesis can be detected by disaccharide analysis measured by anion-exchange chromatography in urine of patients with D4ST1- or DS-epi1-deficient mcEDS. The lack of urinary DS disaccharides is specific for mcEDS [84,98].

**Serum measurements**—Western blot or enzyme-linked immunosorbent assay (ELISA), developed to detect serum levels of TNX, showed reduced TNX levels in clEDS patients with biallelic variants in the *TNXB* gene. These tests are not routinely available since it depends on the availability of specific antibodies [58]. More recently, TNX serum levels were determined in clEDS1 patients using nano-liquid chromatography tandem mass spectrometry (nano-LC/MS-MS) [99].

Western blot analysis of bikunin, a CS-proteoglycan that is abundantly found in blood, showed significantly decreased amounts of bikunin-CS and increased levels of GAG-free bikunin in serum from patients with spEDS-*B4GALT7* and spEDS-*B3GALT6* [100,101].

Although these serum analyses are sensitive enough to detect the respective EDS types, they are, at present, not routinely available in a diagnostic setting. The classic biochemical derangements observed in IEM, such as metabolic acidosis, hyperammonemia, ketosis or hypoglycemia, are absent in EDS.

### 4.2. Ultrastructural Collagen Fibril Analyses

Fixed skin biopsies of EDS patients can be processed for TEM studies, which allow ultrastructural analysis of collagen fibrils. Whereas dermal collagen fibrils are usually perfectly round on cross-section and tightly packed, a range of alterations can be observed in EDS patients (Figure 3). Abnormally shaped collagen fibrils, such as irregularly contoured fibrils, so-called (cauli)flower-like fibrils, are seen in cEDS, but occasionally also in mcEDS-*CHST14*, spEDS-*B3GALT6* or clEDS2, and fibrils with a hieroglyphic appearance occur in dEDS [10,29,67,102]. 

In addition, morphometric analysis showed variable fibril diameters in several EDS types and a shift to larger fibril diameters in clEDS type 2 [67]. Disturbed fibril organization with increased interfibrillar spacing is observed in clEDS type 1, mEDS, mcEDS and spEDS. These ultrastructural findings can be indicative of abnormal collagen fibrillogenesis, but are not specific to EDS, and their presence can support but not confirm an EDS diagnosis.

## Figures and Tables

**Table 1:** Overview of the EDS types, genes and proteins, and inheritance pattern (IP) as defined by the 2017 International EDS Classification with indication of the estimated prevalence, the associated pathophysiological mechanisms and available biochemical tests. AD: autosomal dominant, AR: autosomal recessive, NA: not available, ?: unknown.

EDS Type	Gene	Protein	IP	Estimated Incidence/Reported Individuals	Pathophysiological Mechanism	Biochemical Testing
Defects in Collagen Structure and Collagen Processing						
Classical (cEDS)	*COL5A1* *COL5A2* (*COL1A1* p.(Arg312Cys))	α1-chain of type V procollagen α2-chain of type V procollagen α1-chain of type I procollagen	AD	1:20,000	Decreased type V collagen amounts affecting the initiation and assembly of heterotypic type I/V collagen fibrils Decreased type V collagen amounts affecting the initiation and assembly of heterotypic type I/V collagen fibrils Local destabilization of the type I collagen molecules and production of α1(I) dimers	(Pro)collagen biochemistry
Vascular (vEDS)	*COL3A1* (*COL1A1* p.(Arg312Cys), p.(Arg574Cys), p.(Arg1093Cys))	α1-chain of type III procollagen α1-chain of type I procollagen	AD	1:50,000–1:200,000	Decreased type III collagen amounts affecting the initiation and assembly of heterotypic type I/III collagen fibrils Local destabilization of the type I collagen molecules and production of α1(I) dimers	(Pro)collagen biochemistry (Pro)collagen biochemistry
Arthrochalasia (aEDS)	*COL1A1* *COL1A2*	α1-chain of type I procollagen α2-chain of type I procollagen	AD	<60 reported individuals	(Partial or complete) deletion of exon 6 leading to partial processing of type I procollagen with retention of the N-propeptide of either the pro-α1(I)- or the pro-α2(I)-chain	(Pro)collagen biochemistry (Pro)collagen biochemistry
Dermatosparaxis (dEDS)	*ADAMTS2*	A Disintegrin And Metalloproteinase with Thrombospondin Motifs 2 (ADAMTS-2)	AR	15 reported individuals (14 families)	Absent N-propeptide cleavage of both pro-α(I)-chains	Procollagen biochemistry
Cardiac-valvular (cvEDS)	*COL1A2*	α2-chain of type I procollagen	AR	6 reported individuals (5 families)	Total absence of pro-α2(I)-chains leading to the formation of α1(I) homotrimers	(Pro)collagen biochemistry
Defects in Collagen Folding and Collagen Cross-Linking						
Kyphoscoliotic (kEDS)	*PLOD1* *FKBP14*	Lysyl hydroxylase 1 (LH1) FK506 Binding Protein, 22 kDa (FKBP22)	AR	<100 reported individuals <35 reported individuals	Deficient post-translational hydroxylation of lysyl residues causing impaired crosslink formation in the collagen triple helix Deficiency of the molecular chaperone (possibly) leads to premature interaction and accumulation of collagen molecules in the endoplasmic reticulum	(Pro)collagen biochemistry Urinary crosslink analysis: increased LP/HP ratio (between 2–9)
Defects in Extracellular Matrix Bridging Molecules						
Classical-like (clEDS)	*TNXB*	Tenascin X (TNX)	AR	<65 reported individuals	Interference with the normal organization and mechanical properties of collagen fibrils in the ECM	TNX serum levels
Myopathic (mEDS)	*COL12A1*	α1-chain of type XII procollagen	AD AR	<20 reported individuals	Interference with the normal organization and mechanical properties of collagen fibrils in the ECM	NA
Defects in Intracellular Processes						
Brittle cornea syndrome (BCS)	*ZNF469* *PRDM5*	Zinc Finger Protein 469 (ZNF469) PR/SET Domain 5 (PRDM5)	AR	<55 reported individuals <35 reported individuals	Disturbed ECM regulation, but exact pathophysiological mechanism remains unclear Disturbed ECM regulation, but exact pathophysiological mechanism remains unclear	NA NA
Spondylodysplastic (spEDS)	*SLC39A13*	Zrt/Irt-Like Protein 13 (ZIP13)	AR	13 reported individuals (7 families)	Generalized underhydroxylation of lysyl and prolyl residues of collagen and abnormal crosslinking of collagen in the ECM, but exact pathophysiological mechanism remains unclear	Urinary crosslink analysis: increased LP/HP ratio (around 1)
Defects in Glycosaminoglycan Biosynthesis						
Musculocontractural (mcEDS)	*CHST14* *DSE*	Dermatan 4-*O*-sulfotranferase-1 (D4ST1) Dermatan sulfate epimerase-1 (DS-epi1)	AR	<70 reported individuals	Defective biosynthesis of dermatan sulfate (DS) resulting in depletion of DS	Urinary disaccharide analysis Urinary disaccharide analysis
Spondylodysplastic (spEDS)	*B4GALT7* *B3GALT6*	Galactosyltransferase I (b4GalT7) Galactosyltransferase II (b3GalT6)	AR	<15 reported individuals <50 reported individuals	Absence of the first galactose residue of the tetrasacharide linker region of proteoglycans Absence of the second galactose residue of the tetrasacharide linker region of proteoglycans	Serum bikunin analysis Serum bikunin analysis
Defects in the Complement Pathway						
Periodontal (pEDS)	*C1S* *C1R*	Complement C1s (C1s) Complement C1r (C1r)	AD	<150 reported individuals	Gain of function variants possibly leading to abnormal interactions with components of the ECM Gain of function variants possibly leading to abnormal interactions with components of the ECM	NA
Molecularly Unresolved						
Hypermobile (hEDS)	?	unknown	?	?	?	NA
Novel Type of EDS (Identified After the 2017 Classification)						
Classical-like II (clEDS II)	*AEBP1*	Adipocyte enhancer-binding protein 1 (AEBP1)	AR	9 reported individuals (9 families)	Interference with normal collagen fibril formation, but exact pathophysiological mechanism remains unclear	NA

**Table 2:** Clinical signs and symptoms of the different EDS types. Major clinical criteria according to the 2017 International EDS Classification are written in bold, minor clinical criteria are written in normal font and additional phenotypic features are written in italics. (G)JH: (generalized) joint hypermobility, MVP: mitral valve prolaps.

EDS Type	Integumentary System	Skeletal System	Neuromuscular	Craniofacial	Ophthalmological	Vascular	Cardiac	Other
cEDS (*COL5A1*; *COL5A2*; *COL1A1* p.(Arg312Cys))	**skin hyperextensibility with atrophic scarring**, easy bruising soft doughy skin skin fragility, molluscoid pseudotumours, subcutaneous spheroids, hernia (or history thereof)	**GJH**, complications of JH	*(mild) muscle hypotonia, delayed motor development*	epicanthal folds		*Rarely aortic root dilatation, rarely arterial dissection/rupture*	*MVP (non-progressive)*	
vEDS (*COL3A1*; *COL1A1* p.(Arg312Cys, p.(Arg574Cys), p.(Arg1093Cys)	bruising unrelated to identified trauma and/or in unusual sites, translucent skin, acrogeria	talipes equinovarus, congenital hip dislocation, small joint hypermobility, tendon and muscle rupture		characteristic facial features (large eyes, periorbital pigmentation, small chin, sunken cheeks, thin nose and lips and lobeless ears), gingival recession and gingival fragility	*keratoconus*	**arterial rupture at young age, carotid-cavernous sinus fistula**, early-onset varicose veins		**spontaneous sigmoid colon perforation, uterine rupture during third trimester of pregnancy**, spontaneous pneumothorax
aEDS (*COL1A1*; *COL1A2*)	**skin hyperextensibility**, tissue fragility including atrophic scars, easy bruising, *umbilical hernia*	**congenital bilateral hip dislocation, severe generalized JH with multiple dislocations**, kyphoscoliosis, radiologically mild osteopenia, *fractures, foot and hand deformities, pectus deformity*	muscle hypotonia, *delayed motor development*	*large fontanelle, frontal bossing, hypertelorism, blue sclerae, epicanthal folds, depressed nasal ridge, midface hypoplasia, micrognathia, dentinogenesis imperfecta*				*pregnancy-related complications (breech, PPROM, polyhydramnios, decreased fetal movements)*
dEDS (*ADAMTS2*)	**extreme skin fragility with congenital or** **postnatal tears, progressively redundant, almost lax skin with excessive skin folds at wrists and ankles, increased palmar wrinkling, severe bruisability with risk of subcutaneous haematoma, umbilical hernia**, soft and doughy skin texture, skin hyperextensibility, atrophic scars, hirsutism	**postnatal growth retardation with short limbs**, GJH, osteopenia, *fractures*	delayed motor development	**large** **fontanel, puffy eyelids, excessive peri-orbital skin, downslanting palpebral fissures, blue sclerae, hypoplastic chin**, tooth abnormalities	refractive errors, strabismus, *glaucoma*	*Intracerebral hemorrhage*		**perinatal complications related to tissue fragility**, complications of visceral fragility, *preterm birth (PPROM),*
cvEDS (*COL1A1; COL1A2*)	**skin involvement**, inguinal hernia	**JH**, pectus deformity, joint dislocations, foot deformities			*blue sclerae, refractive errors*		**severe progressive cardiac valvular insufficiency**	
kEDS (*PLOD1*; *FKBP14*)	skin hyperextensibility, easy bruising, umbilical or inguinal hernia PLOD1: skin fragility FKBP14: hyperkeratosis follicularis, *umbilical skin redundancy*	**congenital or early-onset kyphoscoliosis, GJH with (sub)luxations**, osteopenia/osteoporosis, pectus deformity, marfanoid habitus, talipes equinovarus	**congenital muscle hypotonia**, *delayed motor development* FKBP14: muscle atrophy	blue sclerae PLOD1: characteristic craniofacial features: low-set ears, epicanthal folds, down-slanting palpebral fissures, synophrys and high palate	refractive errors PLOD1: microcornea	rupture/aneurysm of medium-sized artery, *antenatal/neonatal brain hemorrhage*		*pregnancy-related complications (breech, PPROM, oligohydramnios, decreased fetal movements)* FKBP14: congenital hearing impairment, bladder diverticula, *learning disabilities*
clEDS-1 (*TNXB*)	**skin hyperextensibility with velvety skin texture and absence of (extensive) atrophic scarring, easily bruisable skin/spontaneous ecchymoses**, acrogeric hands	**GJH**, foot deformities, mallet fingers, clino- or brachydactyly, *(sub)luxations*	mild proximal and distal muscle weakness, axonal polyneuropathy, atrophy of muscle in hands and feet	*narrow/high arched palate*		*rarely arterial aneurysms*	*valvular abnormalities*	oedema in legs in absence of cardiac failure, vaginal, uterine or rectal prolapse, *gastrointestinal complications (perforation, diverticular disease, …), postpartum hemorrhage*
clEDS2 (*AEBP1*)	**skin hyperextensibility with atrophic scarring**, *translucent skin, easy bruising, hernia, aged appearance*	**GJH, foot deformities, early-onset osteopenia**, *joint dislocations (mostly hip), arachnodactyly, (kypho)scoliosis, osteopenia*		*bad tooth quality, dental abnormalities, high narrow palate,*		*varicose veins, aorta dilatation*	*MVP*	*bowel rupture*
mEDS (*COL12A1*)	soft, doughy skin, atrophic scars, *hypertrophic scars*	**proximal joint contractures, (general/distal) JH**, *congenital hip dislocation, congenital (kypho)scoliosis, pectus deformity*	**congenital muscle hypotonia and/or muscle atrophy**, motor developmental delay, myopathy on muscle biopsy					
BCS (*ZNF469*; *PRDM5*)	soft, velvety and/or translucent skin, *mild skin hyperextensibility, easy bruising, hernia*	developmental dysplasia of hip, scoliosis, arachnodactyly, hypermobility of distal joints, pes planus, hallux valgus, mild finger contractures, *fractures, osteopenia/osteoporosis*	hypotonia in infancy (usually mild)	blue sclerae, *frontal bossing, high palate, depressed nasal bridge and/or prominent chin*	**thin cornea with/without rupture, early-onset progressive keratoconus and/or keratoglobus**, enucleation or corneal scarring because of previous rupture, progressive loss of corneal stromal depth, high myopia, retinal detachment		*MVP (non-progressive)*	deafness (often mixed conductive and sensorineural), hypercompliant tympanic membranes
mcEDS (*CHST14*; *DSE*)	**skin hyperextensibility,** **easy bruising, skin fragility with atrophic scars, increased palmar wrinkling**, large subcutaneous hematomas, *hernia*	Recurrent/chronic dislocations, pectus deformities, spinal deformities, peculiar fingers, progressive talipes deformities, *mild postnatal growth restriction, marfanoid habitus*	**congenital multiple contractures (typically adduction/flexion contractures and talipes equinovarus)**, *hypotonia, motor developmental delay, ventricular abnormalities on brain imaging, tethered spinal cord*	**large fontanelle, short downslanting palpebral fissures, blue sclerae, hypertelorism, short nose with hypoplastic columella, low-set and rotated ears, long philtrum with thin upper lip vermillion, small mouth and hypoplastic chin**, *crowded teeth*	strabismus, refractive errors, glaucoma, *retinal detachment*		*congenital heart defects (typically ASD), valve abnormalities, aortic root dilatation*	chronic constipation, colonic diverticulae, pneumo(haemo)thorax, nephrolithiasis/cystolithiasis, hydronephrosis, cryptorchidism in males, *hearing impairment, constipation, diverticula, poor breast development in females*
spEDS *(B3GALT6*; *B4GALT7*; *SLC39A13)*	skin hyperextensibility, soft and doughy, thin and translucent skin *B4GALT7*: single transverse palmar crease *SLC39A13*: hands with finely wrinkled palms	**short stature (progressive in childhood), bowing of limbs**, pes planus, osteopenia, (characteristic X-ray findings of) skeletal dysplasia, *JH* *B4GALT7*: radioulnar synostosis *B3GALT6*: kyphoscoliosis (congenital or early-onset), JH (generalized or restricted to distal joints), peculiar fingers, osteoporosis with spontaneous fractures *SLC39A13*: tapering fingers, hypermobility of distal joints	**muscle hypotonia (ranging from severe congenital to mild later-onset)**, delayed motor development *B4GALT7*: bilateral elbow contractures *B3GALT6*: joint contractures (congenital or progressive) *SLC39A13*: atrophy of thenar muscles	*B4GALT7*: triangular face, wide-spaced eyes, proptosis, narrow mouth, low-set ears, sparse scalp hair, abnormal dentition, flat face, wide forehead, blue sclerae and cleft palate/bifid uvula *B3GALT6*: midfacial hypoplasia, frontal bossing, proptosis, or prominent eyes, blue sclerae, downslanting palpebral fissures, depressed nasal bridge, long upper lip, low-set ears, micrognathia, abnormal dentition, cleft palate, sparse hair, tooth discoloration, dysplastic teeth *SLC39A13*: protuberant eyes with bluish sclerae, *hypodontia of one or few teeth*	*B4GALT7*: clouded cornea, *refractive errors* *B3GALT6*: *hypermetropia, rarely corneal clouding* *SLC39A13*: *refractive errors*	Rarely aortic aneurysm *SLC39A13*: *varicose veins*	*B3GALT6*: *MVP, congenital heart defects*	cognitive impairment *B3GALT6*: lung hypoplasia, restrictive lung disease
pEDS (*C1R*; *C1S*)	**pretibial plaques**, easy bruising, skin hyperextensibility and fragility, wide or atrophic scarring, hernias, acrogeria, *skin translucency*	JH (mostly distal), *kypho (scoliosis)*		**Severe and intractable early-onset periodontitis, lack of attached gingiva**, marfanoid facial features		prominent vasculature, *rarely arterial dissection/rupture*		**family history of first-degree relative who meets clinical criteria, increased infection rate**
hEDS (genetic defect unknown)	unusually soft or velvety skin, mild skin hyperextensibility, unexplained striae, bilateral piezogenic papules, hernia, mild atrophic scarring,	GJH, arachnodactyly, arm span to height ratio ≥1.05,		dental crowding and high or narrow palate		aortic root dilatation with z score > +2	MVP	pelvic floor, rectal and/or uterine prolapse, positive family history of hEDS
